# Lesser Investigated Natural Ingredients for the Management of Obesity

**DOI:** 10.3390/nu13020510

**Published:** 2021-02-04

**Authors:** Muhammed Majeed, Shaheen Majeed, Kalyanam Nagabhushanam, Muthuraman Gnanamani, Lakshmi Mundkur

**Affiliations:** 1 Sami-Sabinsa Group Limited, Peenya Industrial Area, Bangalore 560058, Karnataka, India; mail.1@sami-sabinsagroup.com (M.M.); muthuraman@sami-sabinsagroup.com (M.G.); 2 Sabinsa Corporation, 20 Lake Drive, East Windsor, NJ 08520, USA; shaheen@sabinsa.com (S.M.); kalyanam@sabinsa.com (K.N.)

**Keywords:** anti-obesity, phytochemicals, energy balance, gut microbiota, leptin resistance, satiety, mechanism

## Abstract

Obesity, an epidemiological disorder, is related to various complications in both the developed and developing world. It epitomizes a crucial risk factor for health, decreasing productivity and life expectancy while increasing health care costs worldwide. Conventional therapies with synthetic drugs or bariatric surgery, associated with numerous side effects, recurrence, and surgical complexity, have been restricted in their use. Lifestyle changes and dietary restrictions are the proven methods for successful weight loss, although maintaining a strict lifestyle is a challenge. Multiple natural products have been explored for weight management with varied efficacy. The current review explores less explored natural herbs, their active constituents, and their mechanisms of action against obesity.

## 1. Introduction

Obesity is a challenging condition of excess body fat, caused by an imbalance in energy consumption and expenditure [1]. It is the result of a complex interaction between environment, diet, genetics, lifestyle, endocrine disorders, medication, and psychological factors [2]. As per the World Health Organization (WHO), individuals with body mass index (BMI) of more than 30.0 kg/m^2^ are considered obese, and those with BMI between 25.0 and 30.0 kg/m^2^ are categorized as overweight [3].

Obesity is associated with comorbidities like diabetes, hypertension, hyperlipidemia, cancer, and sleep apnea, and is considered as an independent risk factor for cardiovascular diseases (CVD) [4]. An increase by one unit BMI causes a 4% rise in ischemic risk and a 6% rise in hemorrhagic strokes [5]. Higher systolic and diastolic blood pressure of 3.0 mm and 2.3 mm Hg, respectively, in individuals with 10 kg excess body weight can induce a 12% increased risk of CVD and 24% increased risk of stroke [6]. Besides, obesity is also associated with increased risk for several cancers including colon, endometrial, kidney, esophageal, liver, pancreatic, breast, Hodgkin’s lymphoma, and myeloma [7,8]. According to the WHO, incidence of obesity has tripled since 1975. In a survey conducted in 2016, 1.9 billion adults and 340 million children and adolescents were reported to be obese or overweight [9]. The rapid increase in obesity prevalence and its associated devastating health effects and comorbidities highlight the immediate need for early recognition, control, and treatment of this problem. Although diet control, exercise and lifestyle changes are the fundamental therapeutic options, few drugs have been approved for pharmacotherapy. Several natural products have been widely studied and numerous review articles reference the use of herbs for weight management [10,11,12]. In the present review, we focus on a few of these less explored natural extracts, their phytochemicals, and their mechanism of action in controlling obesity.

## 2. Pharmaceutical Drugs for Obesity

Several anti-obesity drugs have been evaluated since the beginning of the 20th century. The earliest drugs were the thyroid hormones due to their thermogenic effect, followed by chemicals such as dinitrophenol, mitochondrial uncouplers, amphetamines, and serotonergics as appetite suppressors, which were subsequently withdrawn due to safety concerns [13]. Following this, poly diet pills using the combination of amphetamines and thyroid hormones along with several other ingredients were distributed as rainbow pills for diet control. Though they were highly popular, fatalities associated with their indiscriminate use led to their withdrawal mandated by the US FDA [13]. A list of drugs and their present status is presented in Table 1. The search for novel drugs to reduce appetite and improve glucose metabolism to control obesity and diabetes is being pursued with renewed vigor and attention as the economic burden associated with obesity exceeds US$200 billion in the United States alone and is increasing steadily [14].

## 3. Pathophysiology of Obesity

**Figure 1:**
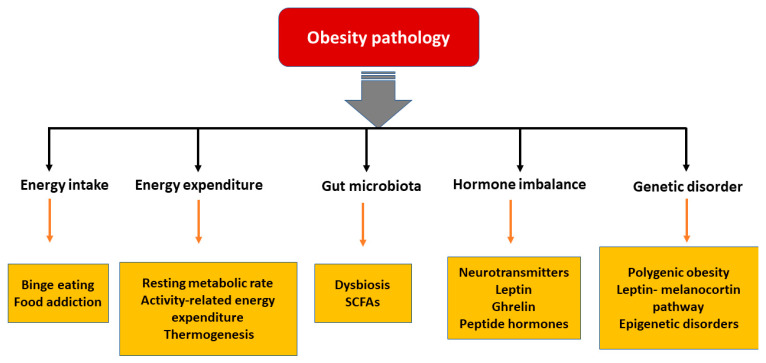
Various parameters affecting the pathology of obesity. [SCFA: short chain fatty acids].

In the last few decades, significant advances have been made in understanding the pathophysiological mechanisms involved in obesity. The neuroendocrinal feedback associated with pathological overeating coupled with physical inactivity seem to be the major factors governing obesity. Apart from this, genetic predisposition, hormonal imbalance, and gut microbial dysbiosis also contribute to accumulation of fat stores [18]. Figure 1 describes the pathophysiological parameters causing obesity.

### 3.1. Energy Intake vs. Expenditure

Obesity can be viewed as an imbalance in energy intake versus energy expenditure. Three components involved in energy expenditure are (i) resting metabolic rate, which is the energy necessary to fuel the body at rest, (ii) activity-related energy expenditure and (iii) diet-induced thermogenesis, which is the energy spent in absorbing and metabolizing food consumed [19]. When an individual ingests more energy than their expenditure, a positive energy balance develops, and this excess energy is converted into triglycerides and stored. When energy intake exceeds energy expenditure by more than 20 kcal/day, 1 kg of fat per year gets accumulated [20]. Thus, a proper balance of energy intake and expenditure is necessary to manage obesity. A complex physiological control system involving signals from the periphery about the status of stored energy, and those that affect energy intake and expenditure, are responsible for maintaining the energy balance [21].

#### Energy Expenditure and Thermogenesis

The adipose tissue is generally considered as a passive depot for the storage of excess calories. Recent studies have shown that it is an active endocrine organ, which takes part in energy balance by releasing free fatty acids, proinflammatory cytokines and adipokines such as leptin and adiponectin, regulating food intake and insulin sensitivity [22]. Adipose tissues are heterogeneous and are classified into white adipose tissue (WAT), which are storage organs, and brown adipose tissue (BAT), which burns energy for thermogenesis. Intermediary beige adipocytes, arising from WAT, have also been described in mammals [23]. Exposure to cold, adrenergic stimulation, and long-term treatment with peroxisome proliferator-activated receptor (PPAR)γ agonists are some of the external cues that induce these beige adipocytes [24]. The brown and the beige adipocytes contain numerous mitochondria and express the uncoupling protein 1 (UCP-1), which regulates energy expenditure, reduces adiposity, and protects experimental animals from diet-induced obesity [25]. Besides UCP-1, brown adipocytes express type 2 iodothyronine deiodinase (DIO2), the transcription coregulators PR domain containing 16, (PRDM16) and Peroxisome proliferator-activated receptor gamma coactivator 1-alpha (PGC-1α), and Cell death activator (CIDE-A), which regulates UCP1 transcription in BAT [26]. With the understanding that BAT can help in energy dissipation, pharmacological interventions targeting browning of WAT are being actively investigated to induce energy balance. Few natural ingredients have been reported to convert WAT to BAT, but Forskolin, Capsaicin, Resveratrol, and Berberine are examples of molecules that could induce browning of WAT [27,28].

### 3.2. Hormonal Imbalance

Neurobiological mechanisms are reported to contribute to eating in the absence of energy demand and hunger. The cortico-limbic system, hypothalamus and hindbrain are three heavily interconnected brain regions that are involved in controlling eating behavior [29]. These are affected by various visual food stimuli under conditions of fasting, weight loss, overfeeding, exercise, hormone infusion, leanness, obesity, and voluntary cognitive control [30]. The important neurostimulators such as serotonin and dopamine play a significant role in food intake. Increased serotonergic signaling is associated with decreased food intake, whereas its decrease induces hyperphagia and weight gain [31]. Likewise, lesser dopaminergic signaling promotes overconsumption of food beyond homeostatic needs [32].

The gut hormones play the most critical role in obesity. The cells of the gastrointestinal tract sense ingested food and release the gut hormones that regulate energy and glucose homeostasis through autocrine, paracrine, and endocrine pathways. Glucagon-like peptide 1 (GLP-1), peptide (PYY) and oxyntomodulin signal the availability of nutrients to the brain and suppress appetite [33]. Additionally, leptin secreted by the adipose tissue acts as a signal for energy availability and promotes satiety. In the absence of food, ghrelin is secreted, which sensitizes the brain to the intake of food [34].

#### Leptin Resistance

**Figure 2:**
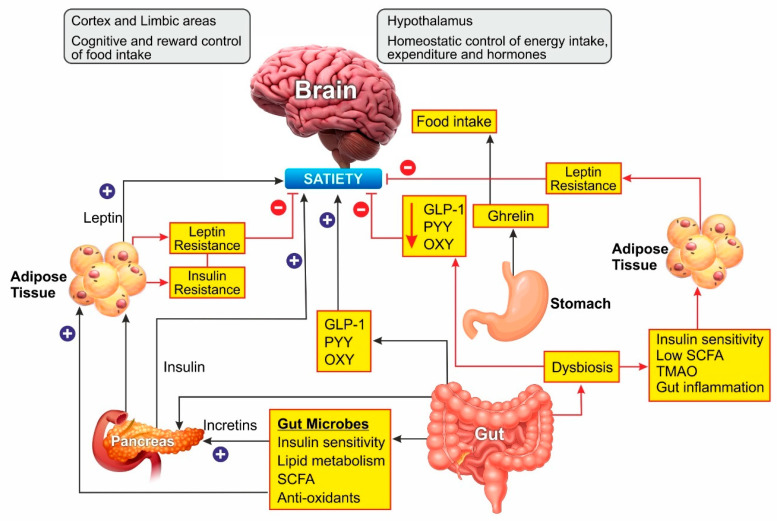
Schematic representation of Gut microbiome and hormonal control of obesity: Ghrelin, produced in the stomach, is a potent stimulator of appetite in the brain. Insulin from the pancreas and leptin from adipose tissue act on the brain to induce satiety. Gut-derived peptides such as GLP-1 augment insulin release from the pancreas. The gut microbiome balances energy homeostasis by releasing short-chain fatty acids with numerous benefits. Dysbiosis of the gut decreases the peptide hormone and increases gut inflammation, which can induce insulin and leptin resistance preventing satiety. In the brain, the hypothalamus region controls energy homeostasis while the cortex and limbal areas are responsible for cognitive reward control of food intake. CCK, cholecystokinin; GLP-1, glucagon-like peptide–1; OXY, oxyntomodulin; PYY, peptide YY; SCFA, short-chain fatty acids. Blue lines represent positive stimulation to control fat storage while red lines represent the causes of obesity.

Leptin is an adipokine that regulates food intake, energy expenditure, immune function, and numerous other physiological activities [35]. Circulating leptin concentrations directly reflect the adipose tissue energy stores and it generally enables energy expenditure while reducing food craving [36]. Leptin binds to its receptor in the brain and mediates its action through the neuroendocrine axes. It also attenuates the hyperglycemia caused by insulin deficiency [37]. Since leptin acts as a messenger for peripheral energy stores, increasing its circulating levels was thought to be a potential treatment for obesity [38]. During the progression of obesity, leptin signaling is affected, leading to leptin resistance. In these cases, the leptin levels are high in serum, but it is unable to bind to its receptor and mediate the physiological activity [39]. Obesity is also associated with leptin resistance, which affects leptin signaling and its downstream physiological effects. Obese patients develop leptin resistance despite high circulating levels of the adipokine, rendering the leptin therapy ineffective. Alleviating leptin resistance is an exciting research area as potential anti-obesity therapy as no drugs are known for this function (Figure 2).

### 3.3. Gut Microbiota

Various bacteria, viruses, fungi, and protozoa colonize the gastrointestinal tract, which is interactively involved in immune, metabolic, and neurological health [40,41]. The gut microbes play a major role in metabolism by fermenting the non-digestible dietary fibers to short-chain fatty acids (SCFA) such as butyrate, propionate, and acetate [42]. These SCFAs are involved in cholesterol metabolism and lipogenesis and are reported to play a central role in appetite regulation [43]. In the last two decades, several studies have shown that probiotic supplements can reduce body weight and improve glucose metabolism in rodents by changing the composition of gut microbiota [44]. Overweight and obese people show a dysbiosis characterized by lower microbial diversity associated with impaired glucose homeostasis and low-grade inflammation [45,46]. A meta-analysis of human clinical trials concluded that obesity was associated with higher counts of Firmicutes, Fusobacteria, Proteobacteria, and *Lactobacillus reuteri*, and lower counts of Bacteroidetes, *Akkermansia muciniphila*, *Faecalibacterium prausnitzii*, *Lactobacillus plantarum*, and *Lactobacillus paracasei*. An increase in the Firmicutes/Bacteroidetes ratio was observed in association with obesity [47], while a reduction in Firmicutes’ proportion with a rise in Bacteroidetes was associated with weight loss [48]. Further, the gut microbiome composition was found to be directly altered by the diet. In animal models, a high-fat diet favored Firmicutes and lowered the Bacteroidetes [49]. The metabolites derived from the fermentation of food by microbiome play a vital role in regulating host metabolism. The gut bacteria convert bile acid in the intestine to deoxycholic acid and lithocholic acid, which stimulate the secretion of incretin hormone GLP-1 and insulin, thereby promoting energy expenditure [50]. Dietary choline metabolism is also linked to microbiome composition. Conversion of choline into trimethylamine-N-oxide (TMAO) by microbiome has been associated with atherosclerosis and metabolic disorders [51]. The conversion of choline to the intermediate trimethylamine is mediated by several intestinal-resident bacteria. The SCFAs produced by gut bacteria are involved in insulin signaling associated with fat accumulation, modulate the secretion of GLP-1 and suppress the inflammatory immune response in the gut [45,52,53]. Inflammation and gut permeability are other markers associated with adiposity [54,55]. These two factors are interlinked as an increased permeability allows bacterial metabolites to leak into the circulation causing low-grade inflammation, a characteristic feature of obesity and insulin resistance [56]. The proinflammatory cytokines, in turn, can cause intestinal barrier disruption [57].

### 3.4. Genetic Predisposition

Mutation in the leptin-melanocortin pathway [58,59], polygenic obesity [60] and epigenetic disorders like Prader-Willi and Temple syndrome [61] play major roles in the pathogenesis of obesity. Some single gene mutations which disrupt the regulatory system of appetite and weight are described in Table 2. The genetic predisposition to obesity has been reviewed by several authors and is beyond the scope of this manuscript.

## 4. Adipogenesis and Growth of Adipose Tissue

A variety of cell types, including endothelial cells, fibroblasts, pericytes, preadipocytes, macrophages, and other immune cells are present in the adipose tissue. The expansion of adipose tissues occurs by both increase in their numbers (hyperplasia) and size (hypertrophy). The adipocytes differentiate from the mesenchymal stem cells (MSC), by a multistep process, involving several genes, signaling and transcriptional factors. Three well-defined phases are involved in the process of adipogenesis: the commitment of MSC to adipocyte lineage, mitotic clonal expansion, followed by terminal differentiation. The final stages of differentiation involve the expression of genes and transcriptional factors such as PPARγ, CCAAT/enhancer-binding proteins (C/EBPs), increase in lipogenesis, and the induction of lipogenic genes such as acetyl CoA carboxylase (ACC), fatty acid synthase (FAS) and adipocyte fatty acid binding protein (aP2) [69]. The canonical Wnt/catenin, Hedgehog and transforming growth factor beta (TGF-β) 1 and 2, Sirtuin (Sirt) 1, microRNA (MiR)-27a and MiR-93, signaling pathways inhibit PPARγ, and C/EBPα expression. The expression of these genes is promoted by the glucocorticoid, cAMP, and bone morphogenic proteins (BMPs) signaling. The microRNA signals by MiR-210 and MiR-146, bromodomain-containing protein 4 (BRD4), Sirt 7 mediated signals also promote PPARγ and C/EBPα expression [70]. The white and brown adipose cells have a distinct origin and morphological characters. The WAT is differentiated from the mesenchymal precursor cells while the origin of BAT is the Myf5 expressing precursors [71]. Further, the brown and white adipocytes store and metabolize lipids in different manners. White adipocytes accumulate nutrient-derived triglycerides and release them by lipolysis during periods of fasting, whereas brown adipocytes oxidize their lipid stores in an elegant heat-producing pathway mediated essentially by UCP1. Recent studies have shown physiological plasticity in WAT. Cold exposure, or treatment with β-adrenergic receptor (β3) agonists that enhance lipolysis, induces a subset of UCP1 positive in WAT, which share additional characteristics with brown adipocytes [72]. Unlike classical BAT these brown-like cells in WAT are not derived from Myf5 positive precursors.

## 5. Herbal and Dietary Supplementation for Weight Management

Traditional and complementary medicine has become an indispensable part of the healthcare system all over the world. In recent decades natural products have come into focus, for their potent pharmacological activities with minimal adverse effects. A variety of natural plants and other natural dietary products have been reported to have anti-obesity activity. There are reportedly more than 54 plant families whose species have shown an anti-obesity potential, the major phytoconstituents being the flavonoids and polyphenols followed by terpenoids, alkaloids and organic acids. These phytochemicals have been reported to regulate fatty acid oxidation, reduce plasma lipid levels, and inhibit pancreatic lipase activity. Few of these phytochemicals have been extensively studied for their molecular mechanism of action [12]. They have been shown to reduce the oxidative stress and systemic inflammation induced by obesity and reduce adipogenesis by inhibiting cell cycle and AMPKα signaling [73,74]. In the next sections we describe the studies related to the mechanism of action of lesser explored extracts and their phytochemicals in relation to the pathophysiology of obesity.

### 5.1. Coleus forskohlii

**Figure 3:**
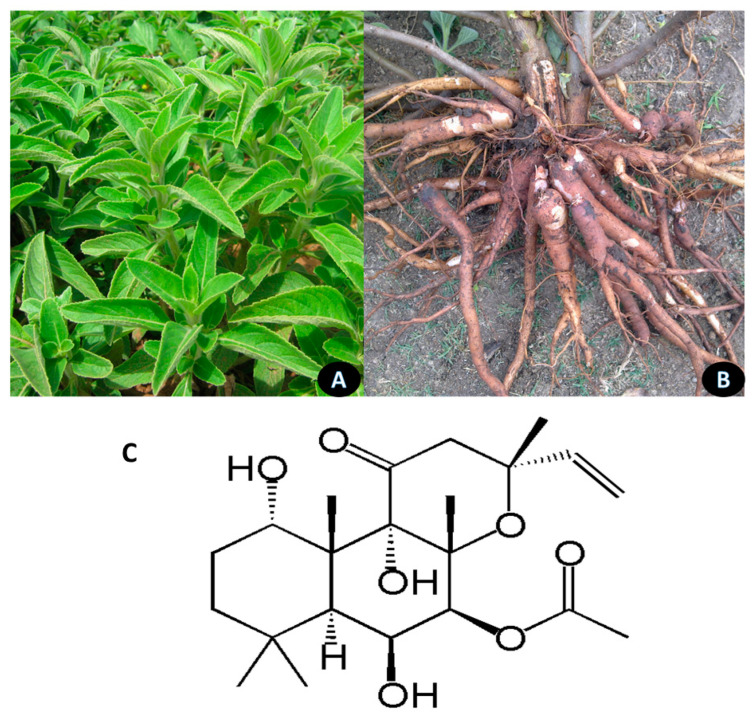
*Coleus forskohlii*. (**A**) leaves; (**B**) roots; (**C**) Forskolin.

Forskolin is a diterpenoid from the root of *Coleus forskohlii,* a native Indian plant of the *Lamiaceae* family. It stimulates adenylate cyclase enzyme, producing cellular cyclic adenosine monophosphate (cAMP), the secondary messenger having a broad range of activities [75]. cAMP induces biochemical events that trigger the metabolic processes and diet-induced thermogenesis, increase lean body mass, and stimulate the loss of body fat [76]. The leaves, roots and structure of Forskolin are shown in Figure 3.

Several clinical and pre-clinical studies report the effect of forskolin in promoting lean body mass and decreasing body fat. In one of the earlier clinical studies, fourteen overweight volunteers, were administered forskolin at a dose of 125 mg twice a day for 12 weeks. The total body weight and body fat decreased significantly, and BMI improved at the end of the study [77]. In an 8 week open labelled study, 6 overweight, women receiving 500 mg of the *C. forskohlii* extract, equivalent to 50 mg forskolin per day, showed significant reduction of body weight and fat content and an increase in lean body mass [78]. In 41 obese patients administered with forskolin along with 250 mg of C. forskohlii extract for 12 weeks, body weight differences were not significant, but changes in hip and waist circumference were significant, suggesting a decrease in fat mass and an increase in bone mass [79]. Similar results were observed in other randomized trials in obese men and women [80,81,82].

Mechanism of action: Forskolin increases the production of Hormone-sensitive lipase (HSL) by the action of cAMP. HSL releases stored triglycerides for metabolic consumption, thus reducing fat storage [83]. The increased cAMP activates the downstream protein kinase A. This cAMP/PKA dependent pathway leads to the overexpression of UCP1, the protein involved in adipocyte browning. PKA activates the p38 mitogen-activated protein kinase (MAPK), which stimulates the expression of the PPARγ coactivator-1α (PGC-1α) and the activating transcription factor 2 increasing UCP1 expression. Alternately, cAMP response element binding protein directly binds on UCP1 promoter in a p38 MAPK-independent manner to induce UCP1 expression [84]. Forskolin also activates AMPK by phosphorylating the tyrosine-172 residue, required for lipolysis activity [85].

### 5.2. Garcinia cambogia and Garcinia indica

**Figure 4:**
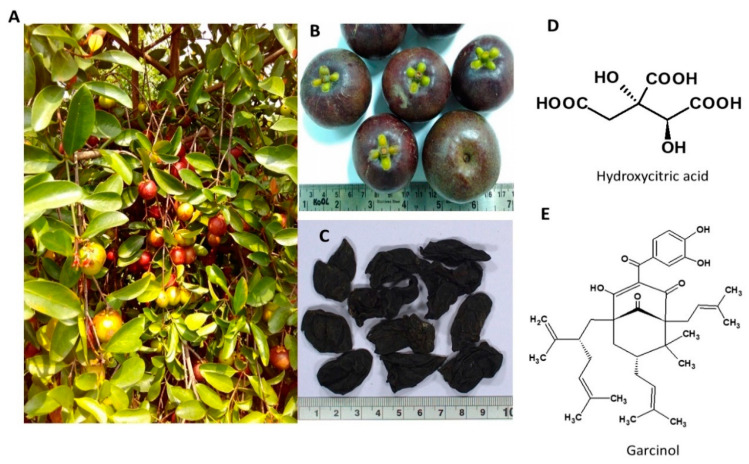
*Garcinia indica*. (**A**) Habitat of the Plant; (**B**) Ripe fruits; (**C**) Dried fruit rinds; (**D**) Hydroxy citric acid; (**E**) Garcinol.

Garcinia, commonly known as kokum, belongs to the Clusiaceae family and is traditionally used as a flavoring agent (Figure 3). For centuries it has been used for culinary purposes in place of tamarind or lemon and as a pharmaceutical and nutraceutical in many regions of South India [86]. The major metabolites in *G. indica* are hydroxy citric acid (HCA), malic acid, citric acid, and tartaric acid while benzophenones, bioflavonoids, xanthones and anthocyanin pigments are the secondary metabolites in the fruit rind [87,88]. Garcinol, Isogarcinol, and Isoxanthochymol are some of the major derivatives of poly-isoprenylated-benzophenones isolated from fruits, dry rinds and leaves [89,90]. As an anti-obesity agent, HCA is reported to reduce food intake, increase energy expenditure, suppress fatty acid synthesis, and enhance glycogen synthesis in the liver [91]. The habitat of the plant and the structures of its phytochemicals are presented in Figure 4.

Garcinol is a yellow crystalline poly-isoprenylated benzophenone derivative extracted from fruit rind of *Garcinia indica* [92]. The extract of *G. indica* standardized for 20% *w*/*w* Garcinol inhibited the differentiation of 3T3-L1 preadipocytes in vitro by downregulating the adipogenic genes and by increasing the transcripts associated with energy metabolism and adipocyte browning [93]. In the animal model, Garcinol induced a dose-dependent reduction of total body weight and visceral adipose tissue in high-fat diet- (HFD)-induced obese mice. Garcinol mediated the anti-obesity activity by reducing endoplasmic stress in preadipocytes and adipose tissues. The results suggested a novel molecular mechanism of Garcinol’s action, acting on the AMPK-ER stress axis [93]. Lee et al. reported a similar result, wherein Garcinol administration to HFD-fed mice showed a reduction in glutamate pyruvate transaminase, total cholesterol, and triacylglycerol. In this study, Garcinol reversed gut dysbiosis by decreasing the Firmicutes-to-Bacteroidetes ratio and controlled inflammation by increasing intestinal commensal bacteria, *Akkermansia muciniphila,* which has been strongly and negatively correlated with age and HFD feeding in mice [94]. In obese mice, a reduction in the *A. muciniphila* could be correlated with expression of inflammation markers, lipid metabolism, circulating glucose, insulin, triglycerides, and leptin levels [95]. In another study, *Garcinia cambogia* extract along with probiotics was observed to change the gut microbial community compared to the probiotic alone. The combination increased the *Bifidobacteria* and decreased *Clostridium aminophilum*, and other strains associated with diet induced obesity [96].

The organic acids, i.e., hydroxy citric acid (HCA) present in Garcinia, are also identified as potential weight loss supplements. Since HCA and *G. cambogia* are well-covered in numerous articles, they are not included in the present review.

Mechanism of action: AMP-activated protein kinase (AMPK) is a major regulator of energy metabolism Once activated, AMPK shifts the cellular metabolism from anabolic pathways to catabolic pathways. Activation of AMPK in adipocytes leads to a decreased fatty acid uptake, decreased triglyceride synthesis, and increased fatty acid oxidation. Garcinol activates the AMPK, and reduces cellular ER stress thereby improving metabolism. Garcinol was also reported to activate PGC-1α, PRDM16, and BMP-7, all involved in increasing UCP1 expression and in browning of white adipocytes. Furthermore, Garcinol shows profound influence on the gut microbiome, regulating the gut dysbiosis, and inflammation. The increase in *A. mucinifila* could be directly correlated to the anti-obesity activity of the molecule, remodulating the gut microbiome to reduce obesity.

### 5.3. Cyperus rotundus

*Cyperus rotundus* is a perennial herb native to India, used to treat various ailments such as diarrhea, diabetes, pyrosis, inflammation, malaria, bowel disorder, cancer, hypertension, and allergy (Figure 4) [97]. The major organic chemicals isolated from various parts of the *Cyperus* species include quinonoid pigments, sesquiterpenoids, flavonoids and stilbene derivatives [98].

**Figure 5:**
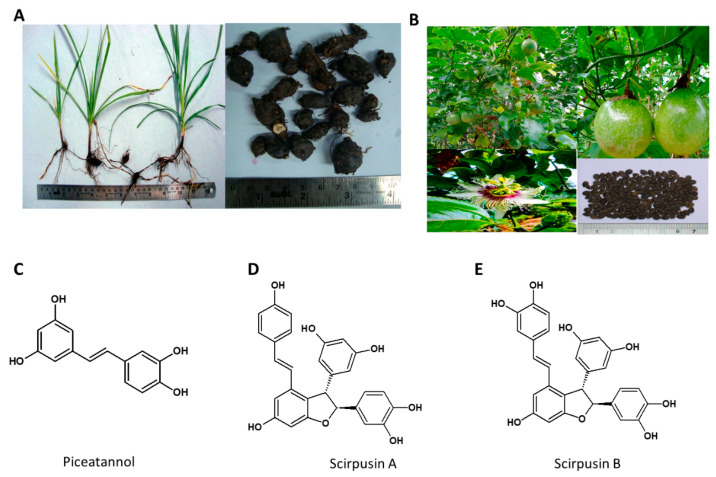
(**A**) *Cyperus rotundus*. Habit of the Plant, and Enlarged view of rhizomes. (**B**) *Passiflora edulis*; habit, enlarged fruit, flower and seeds, (**C**) Piceatannol, (**D**) Scirpusin A, (**E**) Scirpusin B.

Several monoterpenoids, amino acids and fatty acids are also reported in the plant. The ethyl acetate extract of dried, pulverized rhizomes of *C. rotundus* was reported to contain Scirpusin A, Scirpusin B & Piceatannol as major compounds [87]. The chloroform/methyl alcohol extraction of *C. rotundus* yielded novel enantiomeric and meso-stilbene trimers, such as Cyperusphenol A as well as other stilbenoids (Cyperusphenol C & D, Scirpusins A & B, Piceid and Luteolin) [99,100]. The plant rhizomes and the structures of its phytochemicals are presented in Figure 5.

The aqueous tuber extract of C. rotundus at a dose of 100, 200 and 300 mg/kg BW in rats, along with HFD for 40 days, showed a reduction in body weight gain, organ weight, and weight of fat pads. Serum triglycerides, TC, LDL, VLDL, and glucose were reduced while HDL cholesterol increased. The study reported normalization of liver enzymes and the reduction of oxidative stress in HFD obese rats [101]. Similar results were reported with 500 mg/kg BW of ethanolic extract of *c. rotundus* rhizome [102]. The hypolipidemic activity was reported at much lower doses (70, 140, and 280 mg/kg BW) [103]. The ethyl acetate extract of *C. rotundus* rhizome, having 5% total stilbenes with Scirpusin A, Scirpusin B, and Piceatannol as major compounds, showed a dose-dependent reduction of adipogenesis in vitro in 3T3-L1 preadipocyte cells. A reduction in body weight, leptin, corticosteroid concentration, and normalized lipid profile was observed in the HFD induced obese mice (Majeed et al., unpublished work). In a pilot clinical study *C. rotundus* extract showed bodyweight reduction, a significant decrease in waist circumference, and BMI with no adverse events [104]. Piceatannol and its dimer Scirpusin B are also found in passion fruit (*Passiflora edulis*) seeds in larger amounts [105,106]. Piceatannol has been shown to inhibit adipogenesis of preadipocytes 3T3-L1, especially in the first 24 h of adipogenesis by inhibiting the cell cycle progression of preadipocytes. Piceatannol suppressed mitotic clonal expansion by reducing the activation of the insulin-signaling pathways [107]. At a dose of 0.1% and 0.25%, piceatannol significantly lowered body weight, serum cholesterol, and LDL/HDL ratio and had an impact on gut microbiota by increasing the amount of Lactobacillus in high-fat-diet-treated mice [108]. Piceatannol was also reported to induce slight changes in the abundance of *Lactobacillus*, *Clostridium*, and *Bacteroides* in the gut, associated with a decrease in circulating non-esterified fatty acids, LDL-cholesterol and lactate in Zucker rats [109] The molecule also induced mild reno-protective effect in obese Zucker rats by reducing renal fibrosis and lipid peroxidation [110]. In a coculture of adipocyte and macrophage system, piceatannol significantly reduced the release of TNF-α and monocyte chemoattractant protein-1 (MCP-1) [111]. Stilbene, flavonoid and total (poly)phenol intake was associated with higher gut microbiome diversity, an increase in butyrate producing organisms and 20–23% lower prevalence of obesity [112].

Mechanism of action: Piceatannol inhibits adipogenesis by inhibiting mitotic clonal expansion and lowering the protein levels of adipogenic transcription factors such as PPARγ and C/EBPα in 3T3-L1 cells in vitro. They also regulate fatty acid synthase activity to regulate the triglyceride biosynthesis. Piceatannol also regulates the gut microbiome and reduces obesity mediated inflammation.

### 5.4. Curcuma longa

**Figure 6:**
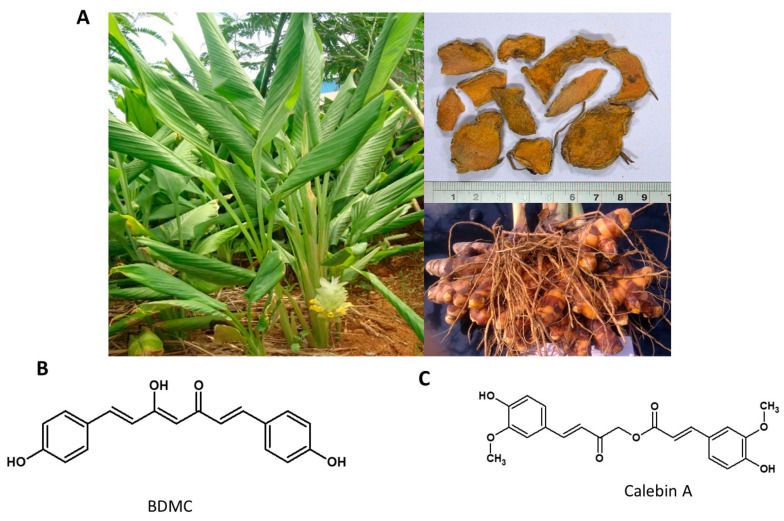
*Curcuma longa*. (**A**) Habit of the Plant; Bunch of fingers; and dried rhizome slice (**B**) Bisdemethoxycurcumin (BDMC), (**C**) Calebin A.

A rhizomatous perennial herb of the ginger family (Figure 5A), it is traditionally used for thousands of years in Ayurveda, Siddha medicine, traditional Chinese medicine, and Unani as a remedy to cure various illnesses related to inflammation, infectious diseases, gastric, hepatic and blood disorders [113,114]. The major ingredients of turmeric powder include 60–70% carbohydrates, 6–13% water, 6–8% protein, 5–10% fat, 3–7% dietary minerals, 3–7% essential oils, 2–7% dietary fiber and 1–6% curcuminoids. It has three major curcuminoids that include, Curcumin 50–60%, Demethoxy-curcumin 20–27%, and Bisdemethoxycurcumin (BDMC) 20–25% [115]. The habitat of the plant and the structures of its phytochemicals are presented in Figure 6.

BDMC is a beta-diketone existing nearly exclusively in its enolic form. It is reported to show a promising anti-tumor property by inducing caspase-dependent and independent apoptosis via the Smad and Akt signaling pathway [116,117]. The anti-obesity effect of BDMC and its mechanism of action was explored in 3T3-L1 mouse adipocytes in vitro and in the diet-induced obesity model in C57BL/6J mice in vivo. BDMC showed potent anti-adipogenic activity at 25 µM concentration. It was reported to suppress adipogenesis by attenuating mitotic clonal expansion by downregulating cyclin A, B, p21 and the mitogen-activated protein kinase (MAPK) signal. Additionally, the adipogenic transcription factors PPAR γ and C/EBP α were also downregulated by BDMC treatment. Similarly, the HFD induced mice treatment with 0.5% dietary BDMC (*w*/*w*) showed significantly lower adipose tissue mass [118].

Curcuma species contain several active phytochemicals with diverse biological activities (Figure 5C). Calebin A, a novel molecule with similar structural features to curcumin but devoid of its iconic 1,3-diketonic feature, was isolated from *C. longa.* It was shown to have anticancer and neuroprotective effects [119,120]. Calebin A belongs to a family of ferulate esters, rightfully called calebenoids, occurring naturally in *Curcuma longa* [121]. These calebenoids, especially Calebin A, seem more stable than Curcumin in a physiological medium with higher chemical stability in acidic and basic media, but sharing several physiological properties with Curcumin in contrast to the noted instability of Curcumin at higher pH values. The Calebenoids seem to arise from a biological Bayer-Villiger type of transformation of curcuminoids [122].

Calebin-A significantly inhibited the differentiation of preadipocytes and lipid accumulation in 3T3-L1 cells in vitro. It suppressed the expression of C/EBPα, C/EBPβ, and PPARγ proteins, which are the master regulators of adipogenesis. Calebin-A effectively decreased weight gain and relative perigonadal, retroperitoneal and mesenteric fat weight in a murine model of diet-induced adipogenesis. Further, Calebin-A reduced hepatic steatosis and restored the liver enzymes to normal levels. Calebin A was reported to mediate its anti-obesity effect through the activation of AMP-activated protein kinase signaling in both in vitro adipocytes and liver tissues [123]. 

Mechanism of Action: BDMC regulates the mitotic expansion of preadipocytes and regulates adipogenesis by downregulating PPAR γ and C/EBP α, while Calebin A mediates its anti-obesity effect by activation of AMPK signaling in both in vitro adipocytes and liver tissues. Calebin A also reduces leptin levels and increases adiponectin, and thus may also act through the adipokine regulation pathway to alleviate leptin resistance and increase metabolism.

### 5.5. Oroxylum indicum

**Figure 7:**
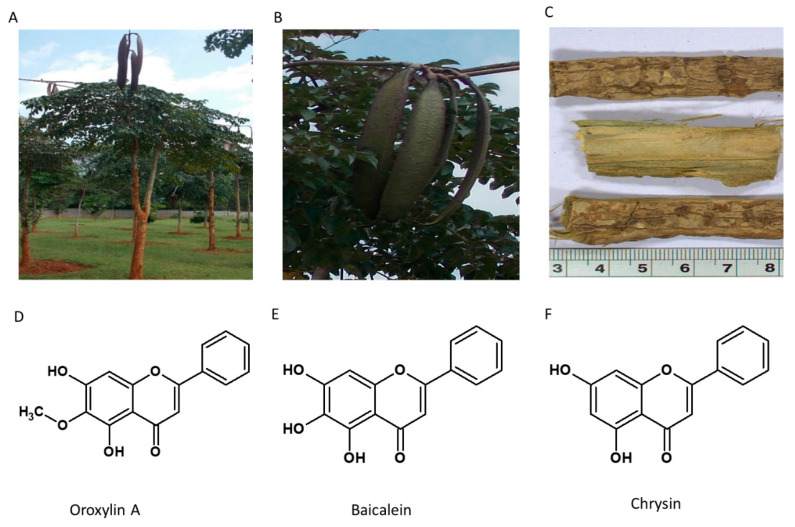
*Oroxylum indicum*. (**A**) Habitat of the plant; (**B**) Fruits; (**C**) Dried bark; (**D**–**F**) Oroxylin A, Baicalein and Chrysin.

Also known as “Sonapatha or Indian trumpet tree,” *Oroxylum indicum* belongs to the Bignoniaceae family (Figure 6). It is commonly found in tropical countries such as India, Japan, China, Sri Lanka, and Malaysia. Various plant segments are reported to be used in traditional medicine for cancer, diarrhea, fever, ulcer, jaundice, and arthritis [124]. The plant contains several secondary metabolites like polyphenols, flavonoids, tannins, terpenoids and alkaloids. It contains an essential oil that gives a specific aroma to the plant [125]. Various active ingredients have been isolated from *O. indicum*. The stem bark contains Oroxylin-A, Chrysin, Baicalein, Scutellarin-7-rutinoside, Tannic acid, Sitosterol, galactose, Baicalin, Biochanin-A and Ellagic acid [126,127]. The habitat of the plant and the structure of the active constituent are given in Figure 7.

Among the several flavonoids, Oroxylin A has been explored for its wide range of therapeutic applications ranging from anticancer, anti-obesity, antioxidant, anti-inflammatory, cardioprotective and neuroprotective roles [128].

The *O. indicum* extract composed of flavonoids, alkaloids, steroids, glycosides, and tannins exhibited a dose-dependent reduction of lipid accumulation in 3T3-L1 cell lines and showed an inhibitory effect on lipase activity [129]. Similarly, the ethyl acetate extract of *O. indicum* bark, containing three bioactive ingredients Oroxylin A, Chrysin and Baicalein, showed around 75% inhibition of lipid accumulation in in vitro cells at 50 µg/mL. The transcription factors PPAR γ and C/EBPα were downregulated by Oroxylin A and Chrysin [130]. The mRNA expression of lipogenic genes controlling adipogenesis, including SREBP-1c, GLUT4, FAS and leptin, was inhibited by *O. indicum* extract containing Quercetin, Apigenin, Kaempferol, Baicalein and Biochanin A [131]. Oroxylin A was also found to be more potent than non-methoxylated flavonoids Morin, Naringenin and Kaempferol in the same study.

Mechanism of Action: *O. indicum* extract containing Quercetin, Apigenin and Kaempferol prevents lipid uptake in the intestine by inhibiting pancreatic lipase and reduces the activity of adipogenic and lipogenic genes controlling adipogenesis, including PPAR*γ*2, SREBP-1c, GLUT4, FAS and leptin, while Oroxylin A and Chrysin regulate adipogenesis by downregulating PPAR γ and C/EBP α.

### 5.6. Pterocarpus marsupium

**Figure 8:**
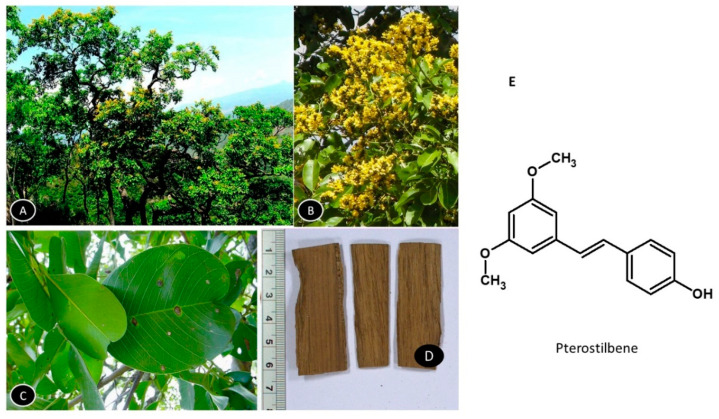
*Pterocarpus marsupium*. (**A**) Habitat of the plant; (**B**) A portion of shoot showing inflorescence and flowers; (**C**) Enlarged view of leaves; (**D**) Wood slices and major actives, (**E**) Pterostilbene.

*Pterocarpus marsupium* is a large deciduous tree, the heartwood extracts of which are extensively used as an antibiotic and hypoglycemic to control blood sugar (Figure 7). The Kino gum obtained from the bark is reported to have astringent, anti-diarrheal, and anti-hemorrhagic properties and the leaves are used to treat skin-related diseases. The tree is found in central and peninsular India. It can survive excessive temperatures in summer and prefers fertile, deep clayey loam soil with good drainage for growth [132]. Pterostilbene is the most actively studied ingredient of the extract [133]. Figure 8 shows the habitat of the plant and the structure of pterostilbene.

Pterostilbene administered at 15 and 30 mg/kg BW for six weeks in obesogenic rats reduced the adipose tissue mass by 15.1% and 22.9%, respectively, which was associated with a reduction in fatty acid synthase. The compound decreased the activity of acetyl-CoA carboxylases involved in regulating the biosynthesis and metabolism of fatty acids and activated the AMPK gene, thus, inducing protection against diet-induced obesity [134]. Pterostilbene was reported to inhibit adipocyte differentiation in 3T3-F442A preadipocytes and eliminate the lipogenic effect of insulin without inhibiting its antilipolytic action and glucose uptake, revealing a unique interaction with adipocytes [135].

Pterostilbene downregulated the expression of adipocyte conditioned medium (aCM)-induced fatty acid-binding protein 5 (FABP5) and pro-metastatic factors such as VEGF, MMP2, MMP9, and TNF-α by inhibiting NF-κB, β-catenin and PPAR-γ. It was also reported to suppress PI3K, Akt, p38 MAPK, ERK and JNK1/2 signaling pathways and to alleviate the adiposity-induced metastasis in obesity-related colorectal cancer cells [136].

The effect of Pterostilbene on brown adipose tissue thermogenic markers was analyzed in Zucker rats. A significant reduction in white adipose tissue and an increase in gene expression of UCP-1, PGC-1α, carnitine palmitoyl-transferase 1b (Cpt-1b), peroxisome proliferator-activated receptor α (PPAR-α), Nrf1 and Cox-2, were observed with oral administration of 15 and 30 mg/kg BW of Pterostilbene for six weeks. The increased expression of these adipocyte browning and anti-inflammatory genes resulted in thermogenesis and increased oxidative capacity in the obese rats [137]. A similar increase in genes associated with adipocyte browning i.e., Cidea, Ebf2, PGC1α, PPARγ, Sirt1, and Tbx1 were observed in HFD-fed mice [138]. In support of these observations, a recent study showed that both Pterostilbene and Resveratrol induce thermogenic activity by enhancing fatty acid oxidation and mitochondrio-genesis [139].

Oral supplementation of Pterostilbene at 15 mg/kg BW for 16 weeks to Zucker rats showed protective anti-obesity effects, improved metabolic function and structural changes in gut microbial composition. The *Akkermansia* and *Odoribacter* genus were modified in the intestine [140].

Mechanism of Action: Pterostilbene decelerates the progression of adipogenesis by decreasing the expression of C/EBPα, and PPARγ suppresses lipogenesis by decreasing fatty acid synthesis. Additionally, it acts by increasing UCP1 and NRF-1 mRNA levels in interscapular BATs from obese rats, as well as by changing the gut microbial profiles.

### 5.7. Withania somnifera

**Figure 9:**
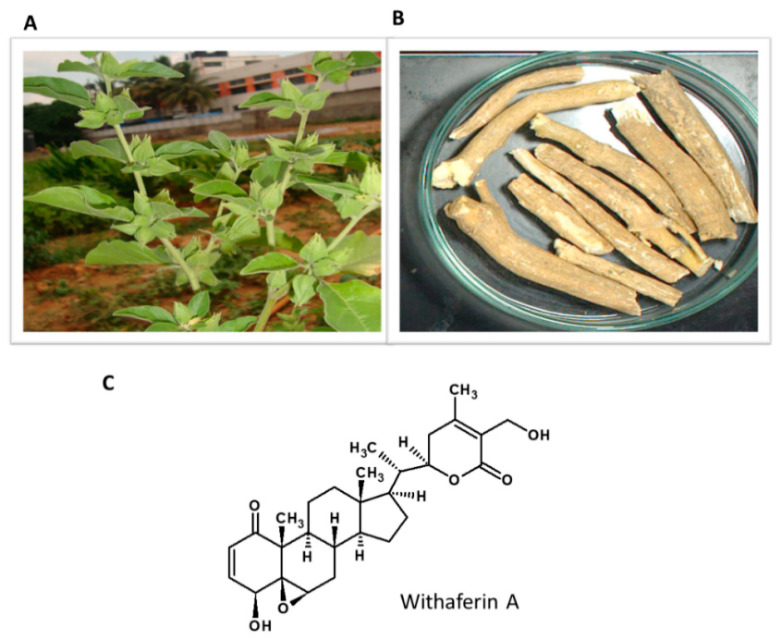
*Withania somnifera*. (**A**) Habitat of the plant; (**B**) Dried stem; (**C**) Withaferin A.

*Withania somnifera* or Ashwagandha (winter cherry) is a well-known medicinal plant in Ayurvedic medicine used for anxiety, depression and to relieve stress (Figure 8). Ashwagandha’s medicinal properties are mainly associated with bioactive ingredients such as withanolides and their glycoconjugates [141]. Withaferin A (WA) is a natural steroidal lactone present in various parts of the plant [142]. Figure 9 shows the habitat of the plant and the structure of the active constituent.

Investigations to identify molecules that can alleviate leptin resistance is an under-explored area for controlling obesity. In an elegant experiment, Liu et.al. used a connectivity map using Endoplasmic reticulum (ER) stress reduction as a tool to identify small molecules which can act as leptin sensitizers, and identified Celastrol, a pentacyclic triterpene extracted from the *Tripterygium wilfordi* plant, as a potent leptin sensitizer [143]. By analyzing a library of small molecules that have mRNA expression profiles similar to that of Celastrol, the same group identified another naturally occurring compound, Withaferin A, as a leptin sensitizer [143]. Withaferin A was reported to be a potent leptin sensitizer, reducing ER stress with additional beneficial effects on diabetes. Obesity induces stress in different organelles and ER stress is one major consequence of excess energy. ER stress has recently been reported to play a central role in leptin resistance [144]. The ER is responsible for secretory protein synthesis and, under excess energy levels, the ER activity is perturbed, leading to an unfolded protein response that aims to reestablish ER homeostasis [145]. Prolonged ER stress causes metabolic, inflammatory, neurodegenerative and cardiovascular diseases [146]. In diet-induced obese mice, treatment with Withaferin A was found to reduce body weight, food intake and hepatic steatosis with beneficial effects on glucose metabolism, independently of its leptin-sensitizing effect [143]. Withaferin A showed only marginal effects in ob/ob and db/db mice which are deficient in leptin signaling. The study suggested that Withaferin A may be a promising candidate for the treatment of obesity. Dietary Withaferin A for 12 weeks, significantly improved hepatic insulin sensitivity, and adipocytokines with enhanced glucose tolerance were seen in HFD induced obese mice. These results were observed along with attenuation of hepatic inflammation, oxidative stress, and insulin resistance in mice [147]. An ethanol extract of WS enriched with Withaferin A was found to suppress increases in bodyweight, serum lipids, and lipid accumulation in the liver and promote browning of subcutaneous fat in mice by increasing mitochondrial uncoupling protein 1 (UCP1) expression [148]. *Withania somnifera* extract was also found to increase energy expenditure by increasing oxygen consumption and thermogenesis in HFD-fed mice.

The overall mechanism action of the herbs and their active ingredients are described in Figure 9.

Mechanism of Action: Withaferin A from *W. somnifera* is a small molecule, which acts as a leptin sensitizer by reducing cellular ER stress. The molecule also reduces oxidative stress and inflammation. Withaferin A enriched extract induces adipocyte browning and increases oxygen consumption and energy expenditure.

**Figure 10:**
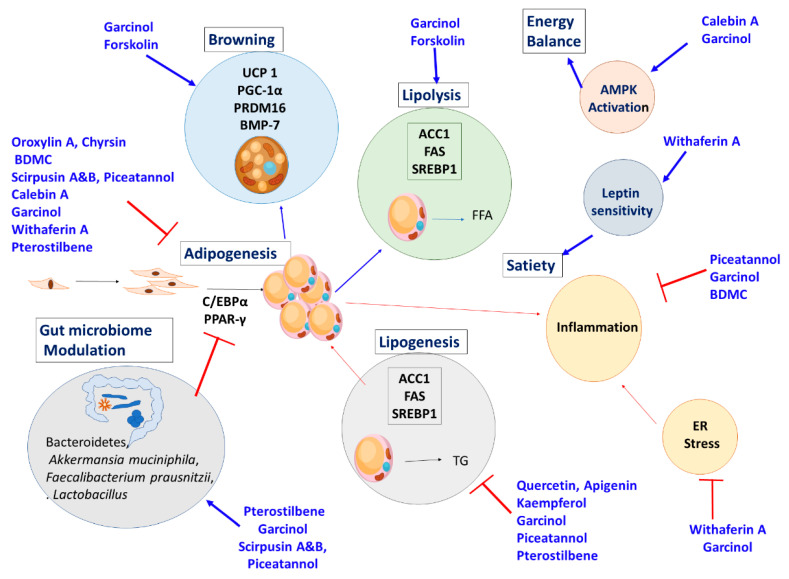
Molecular pathways modulated by the active ingredients in modulating and regulating the obesity related genes and pathways. (**┴**) Inhibited; (**↑**) Promoted.

The mechanism of action of the phytochemicals are given in Figure 10.

## 6. Other Plants and Phytochemicals with Anti-Obesity Activity

Several plant extracts with active phytochemicals have been explored for their anti-obesity activity and the list is exhaustive. Anthocyanins, the pigmented flavonoids, have anti-inflammatory, anti-diabetic and anti-obesity activities. In animals, anthocyanin supplementation reduced body weight gain, visceral fat and lipid levels, and alleviated leptin resistance [149]. Purified cyanidin-3-glucoside was found to induce thermogenesis by increasing mitochondrial UCP1 expression and activating AMPK phosphorylation [149]. Anthocyanins also have a positive interaction with gut microbiome and reduce obesity associated inflammation [150]. The fruits of *Poncirus trifoliata* L. contain several flavanones. Poncirin, one of the major flavanones, was found to have anti-obesity and anti-hyperglycemic effects in HFD included obese mice by modulating lipid and glucose metabolism [151]. 

We have covered a few of the plant extracts and their active phytochemicals with anti-obesity activity and hope that this review will provide a platform to understand the use of phytochemicals to tackle this global health issue.

## 7. Conclusions

Obesity, a multifactorial disorder, is the world’s third-largest prevalent disease. Various pathological features, including energy intake, energy expenditure, genetic disorder, gut microbiota imbalance and hormonal imbalance contribute to its complexity. The physiological pathogenesis, in turn, contributes to various comorbidity disorders affecting multiple parts of the body. To address the same, lifestyle management, surgical and pharmacological therapies are being widely used. The use of natural anti-obesity products may provide an alternative and safer tool in achieving weight-loss goals. Studies using isolated pure compounds and standardized natural extracts to understand the molecular mechanisms of action will enable a more scientific blending of these products for a synergistic activity for weight management. One major advantage of natural products is their multiple actions in controlling the comorbidities associated with obesity. While natural products generally show an effect in vitro and animal models, the major challenge has been in translating these results to clinical data. Future research focusing on clinical studies with well-defined endpoints would enable some of these herbal products to see the light of day in the market for human consumption.

## 8. Patents

US patent 10085963 (Forskolin), 10172903 (Scirpusin A, Scirpusin B and Piceatannol), 9328330 (Calebin A) are patents assigned to Sami Labs limited and Dr Muhammed Majeed for their anti-obesity activity. 

## Figures and Tables

**Table 1:** List of pharmaceutical drugs and their present status. (Adapted from [15,16,17]).

Drug Name	Mechanism of Action	Contraindications and Side Effects	Year Approved and Present Status
Phentermine	Centrally acting sympathomimetic agent, appetite suppressant	Increased blood pressure and heart rate.	1959 Approved for short term
Fenfluramine	Increasing serotonin levels through decreasing reuptake of serotonin	Heart valve damage and major adverse cardiovascular events	(1973–1997) Withdrawn
Dexfenfluramine			(1996–1997) Withdrawn
Sibutramine			(2001–2002) Withdrawn
Orlistat	Inhibiting pancreatic lipase	Hypertension, Diabetes, Hyperlipidemia	1999 Approved
Rimonabant	Selective central cannabinoid (CB1) receptor antagonist	Psychiatric adverse events	(2006–2007) Withdrawn
Lorcaserin	Selective serotonin 2c (5-HT_2c_) receptor agonist	Occurrence of cancer	(2012–2020) Withdrawn
Phentermine-topiramate	Slowing gastric motility and suppressing appetite	Glaucoma, Hyperthyroidism	2012 Approved
Liraglutide (saxenda)	GLP-1 receptor agonist control appetite by mimicking the natural hormone	Medullary thyroid cancer, multiple endocrine neoplasia type 2, C-cell hyperplasia of thyroid, decreased kidney function and pancreatitis	2014 Approved
Semaglutide			Phase III

**Table 2:** Monogenic mutations linked to obesity.

Name	Gene	Chromosomal Position	Action	Reference
Leptin	*LEP*	7q32.1	Secreted by adipocytes and functions as satiety signal in hypothalamus	[62]
Leptin receptor	*LEPR*	1p31.2	Functions as a receptor for leptin to mediate its effect	[63]
Proopiomelanocortin	*POMC*	2p23.2	Its deficiency results in the absence α MSH which regulates appetite	[64]
Melanocortin 4 receptor	*MC4R*	18q21.32	Appetite regulation, binds to α MSH	[65]
Single-minded Drosophila Homologue-1	*SIM1*	6q16.3	Transcriptional factor required for regulating appetite	[66]
Neurotrophic Tyrosine Kinase Receptor Type 2 and Brain Derived Neurotropic factor	*NTRK2, BDNF*	9q21.33, 11p14.1	These neuro-tropins are involved in regulation of food intake and body weight	[67]
SH2B adaptor protein	*SH2B1*	16p11.2	Positive regulator of leptin sensitivity	[68]
